# Medical students’ interest in research: changing trends during university training

**DOI:** 10.3389/fmed.2023.1257574

**Published:** 2023-10-19

**Authors:** Raquel Sanabria-de la Torre, María I. Quiñones-Vico, Ana Ubago-Rodríguez, Agustín Buendía-Eisman, Trinidad Montero-Vílchez, Salvador Arias-Santiago

**Affiliations:** ^1^Biosanitary Research Institute of Granada (ibs.GRANADA), Granada, Spain; ^2^Department of Biochemistry and Molecular Biology III and Immunology, University of Granada, Granada, Spain; ^3^Dermatology Department, Virgen de las Nieves University Hospital, Granada, Spain; ^4^Cell Production and Tissue Engineering Unit, Virgen de las Nieves University Hospital, Granada, Spain; ^5^Dermatology Department, School of Medicine, University of Granada, Granada, Spain

**Keywords:** evidence-based medicine, medical students, research, school of medicine, barriers & facilitative factors

## Abstract

**Introduction:**

Research is an important aspect of medical training and plays a vital role in the advancement of evidence-based medicine. However, little is known about medical students’ attitudes towards research. So, the aim of this study was to assess the opinion of medical students on scientific research.

**Methods:**

A cross-sectional study was designed that included students from the Faculty of Medicine of the University of Granada (UGR), Granada, Spain. A survey was distributed to assess their interest about research during undergraduate studies (1) and following graduation (2), participation in research activities (3), barriers towards research (4), expectation values and self-perceived skills (5). The opinions of students who had not taken clinical subjects (2nd year students) and students who had taken clinical subjects (4th and 6th year students) were compared.

**Results:**

91 students were included in the study (32 were 2nd year students and 59 were 4th and 6th year students). More 4th and 6th year students showed no interest in research (50.4% vs. 28.1%, *p* = 0.042) or in pursuing a doctoral thesis (75% vs. 50.9%, *p* = 0.079) than 2nd year students. In addition, more 4th and 6th year students felt that they did not have sufficient skills to engage in scientific research (52.4% vs. 18.9%, *p* = 0.002). Likewise a greater number of 4th and 6th year students considered that the professors did not encourage scientific research activities (74.6% vs. 40.6%, *p* = 0.002). Generally, students do not participate in scientific dissemination events. The main barriers to research identified were lack of funding and lack of awareness of opportunities.

**Conclusion:**

Interest in research among medical students seems to decrease as the academic years progress. More research promotion could be implemented during the years of university studies.

## 1. Introduction

Medicine and research are inevitably linked, since a better knowledge of human pathophysiology drives therapeutic advances and allows an appropriate bench-to-bedside translation of results (1). The progress of medicine depends fundamentally on the training and productivity of scientists engaged in health sciences research. Research training is essential during medical studies, as the involvement of medical scientists in the field of health research represents a valuable contribution since ultimately physician-scientists are necessary to link basic science research and clinical practice successfully (2). Scientific research plays a crucial role in the education of medical students by providing them with up-to-date knowledge, fostering critical and analytical skills, developing communication skills, and laying the foundation for a future career in medical research, ensuring high quality, evidence-based medical care for patients (1, 3). On the other side, physicians who pursue mainly clinical work require some scientific background to provide the best care, based on the recent research findings and current state of the art (2). In fact, the majority of medical professors attending the 26th Annual Conference of the International Association of Medical Science Educators indicated that they believed scientific education is critical to the development of doctors (4).

Currently, educational institutions in Europe, including the Faculty of Medicine of the University of Granada (UGR) in Spain, provide students with formal and informal opportunities to engage in research and to reduce the likelihood of research misconduct (5). The inclusion of specific courses as educational campaigns in curriculum can be an effective tool to significantly increase the level of awareness among medical students (6). In this way, the UGR has included the compulsory subject “Fundamentals of Health Research and Bioethics” in the curriculum of the Degree in Medicine. The ultimate benefit of these undergraduate experiences may be the promotion of research quality and long-term commitment to good research practices (7). However, it is unclear to what extent undergraduate research is meaningful to students in terms of providing productive and rewarding research experiences (8).

Investigating students’ views on research during medical degree and their expectations regarding their future research careers could help to identify factors that may constitute an effective research curriculum and shed light on whether research is actually promoted in the classroom, as well as identifying the critical points that limit the interest of medical students. The main objective of the present study was to know the perceptions of medical students about scientific research during their university studies as well as following graduation.

## 2. Materials and methods

### 2.1. Study design and population

A cross-sectional study was conducted involving 2nd, 4th, and 6th year medical students at the School of Medicine of UGR during the second semester of the 2022–2023 academic year. The opinions of students who had not taken clinical subjects (2nd year students) and students who had taken clinical subjects (4th and 6th year students) were compared. Participation was entirely voluntary and confidentiality was preserved at all stages of the study, as the survey was anonymous and no personal information was collected or stored.

### 2.2. Instrument

Research in this study is identified with the following activities:

(i) Clinical research: observation and study of patients. This may include monitoring of disease progression, evaluation of treatments and identification of risk factors.
(ii) Epidemiological research: study of disease patterns in populations. This involves the collection and analysis of public health data to identify trends and risk factors.
(iii) Literature review: bibliographic research in which the existing scientific literature on a specific medical topic is analyzed.
(iv) Education research: teaching methods, evaluation of training programmes or development of medical curriculum.

**Figure 1:**
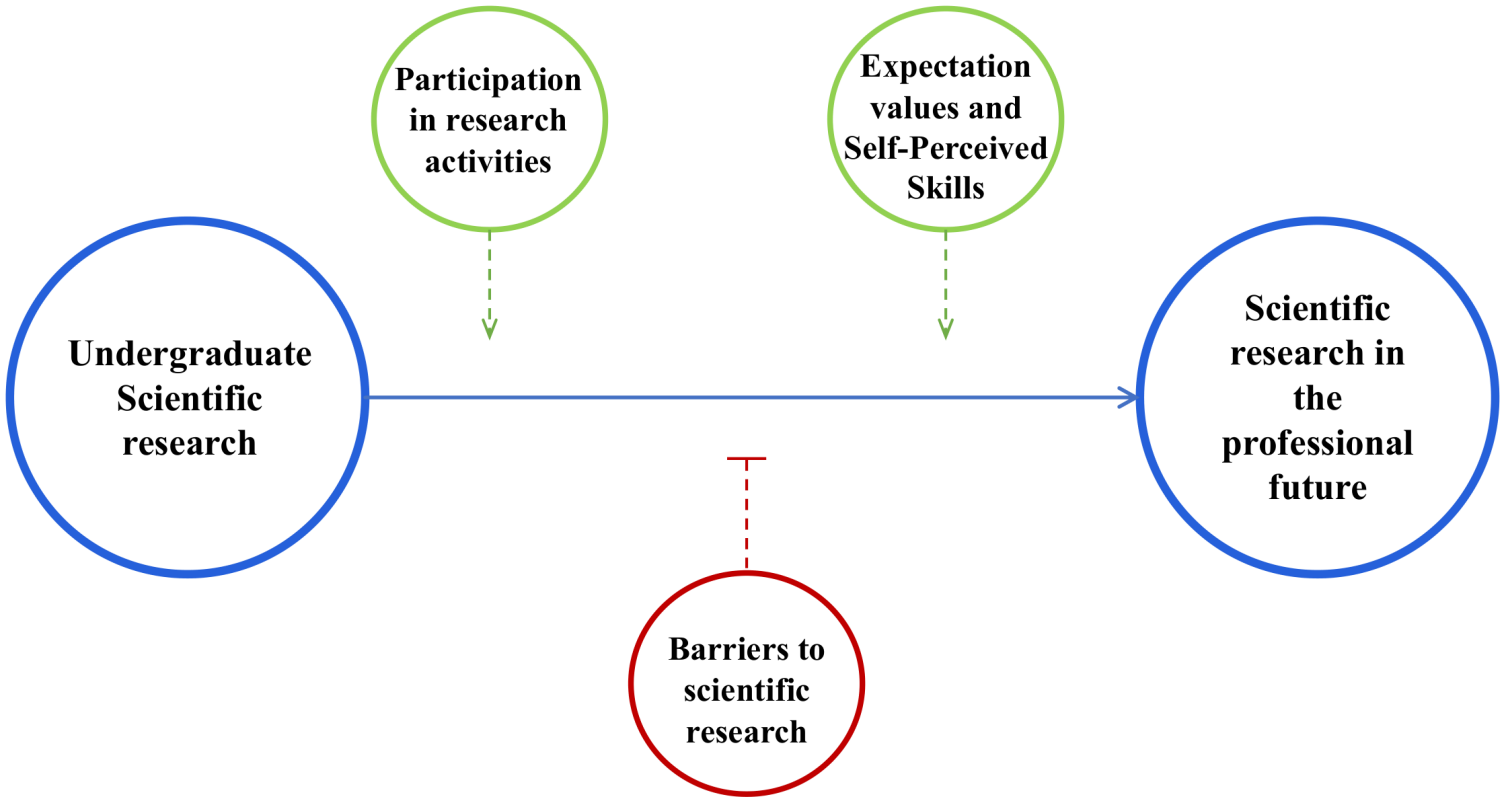
Graphical representation of the five areas related to scientific research included in the survey. Positive (green color) and negative factors (red color) that can influence the scientific vocation at undergraduate or professional future level (blue color) of medical students are considered.

The students received a survey composed of 35 items related to research divided into 5 main sections: (1) Undergraduate Scientific Research (USR; 7 questions); (2) Scientific Research in the Professional Future (SRPF; 9 questions); (3) Participation in Research Activities (PRA; 4 questions); (4) Barriers to Scientific Research (BSR; 7 questions) and (5) Expectation Values and Self-Perceived Skills (EVSPS; 7 questions) (Figure 1).

Prior to the study the survey was assessed by professors and researchers collaborating with the Dermatology Department of UGR. The survey was evaluated in terms of clarity and comprehensibility of the questions and comments and suggestions were used to develop the final version of the survey. Supplementary Table S1 shows the English version of the survey.

### 2.3. Data collection procedure

Students were contacted before lectures or internships and invited to participate in the study. After explaining the objectives of the study, the researchers provided a QR code that directed students to the questionnaire on Google forms. Students who agreed to participate in the study then completed the questionnaire online. Anonymity and confidentiality of the students were guaranteed.

### 2.4. Statistical analysis

A descriptive analysis of the population characteristics was performed. Continuous data were expressed as mean ± standard deviation (SD) and qualitative data as relative (absolute) frequency. The Shapiro–Wilk test was used to determine the normality of the data distribution and Levene’s test was used to test for homogeneity of variance. Student’s T-test or Wilconson rank sum test, depending on the normality of the data, were used to compare continuous variables between students that had not taken clinical subjects (2nd year students) and students who had taken clinical subjects (4th and 6th year students). The Chi-square test or Fisher’s exact test were applied to nominal data when necessary. Significance was set for all tests at two tails, *p* < 0.05. Statistical analyses were performed using JMP version 14.1.0 (SAS institute, North Carolina, United States).

In a reference population of 1,506 medical students, a random sample of 87 individuals is sufficient to estimate, with a confidence of 95% and a precision of ± 15 percentage units, where a population percentage is expected to be around 80%. The percentage of replacements required is expected to be 70%. 121 students were invited to participate in the survey and 91 students responded (75% response rate).

### 2.5. Ethics statement

This study was conducted in accordance with the local legislation and institutional requirements and was approved by the Committee on Ethics in Human Research UGR (CEIH-UGR) under the number 3138/CEIH/2023, dated 30 January 2023. The nature of the study was explained to all participants, who agreed to participate by giving their written electronic consent. The measurements were non-invasive, and the confidentiality of participants’ data was strictly preserved.

## 3. Results

### 3.1. Study population

The study included a total of 91 students, being 76.9% (70/91) females, with an average age of 21.67 ± 2.22 years and an average academic record of 7.87 ± 0.67. 35.2% (32/91) were students that had not taken clinical subjects (2nd year students) and 64.8% (59/91) had taken clinical subjects (4th and 6th year students).

### 3.2. Undergraduate Scientific Research

To analyze the scientific vocation of UGR medical students during their undergraduate studies, a section of 7 questions (USR1-7) was included (Table 1). Among these questions, the importance students attach to research with regard to clinical outcomes, their interest in participating in research projects and the influence of professors on their scientific vocation was approached.

**Table 1:** Survey section 1: Undergraduate Scientific Research, composed of 7 items (USR1-7).

Item	Total					2nd Course					4th and 6th Course					*p* value
	TA	A	N	D	TD	TA	A	N	D	TD	TA	A	N	D	TD	
USR1	23.1% (21/91)	50.6% (46/91)	20.9% (19/91)	4.4% (4/91)	1.1% (1/91)	31.3% (10/32)	56.3% (18/32)	12.5% (4/32)	0.0% (0/32)	0.0% (0/32)	18.6% (11/59)	47.5% (28/59)	25.4% (15/59)	6.8% (4/59)	1.7% (1/59)	0.1905
USR2	0.0% (0/91)	18.7% (17/91)	18.7% (17/91)	36.3% (33/91)	26.4% (24/91)	0.0% (0/32)	25.0% (8/32)	34.4% (11/32)	34.4% (11/32)	6.3% (2/32)	0.0% (0/59)	15.3% (9/59)	10.2% (6/59)	37.3% (22/59)	37.3% (22/59)	0.0017
USR3	16.5% (15/91)	33.0% (30/91)	30.8% (28/91)	12.1% (11/91)	16.5% (15/91)	37.5% (12/32)	43.8% (14/32)	12.5% (4/32)	6.3% (2/32)	0.0% (0/32)	5.1% (3/59)	27.1% (16/59)	40.7% (24/59)	15.3% (9/59)	11.9% (7/59)	<0.0001
USR4	49.5% (45/91)	39.6% (36/91)	8.8% (8/91)	2.2% (2/91)	0.0% (0/91)	59.4% (19/32)	31.3% (10/32)	6.3% (2/32)	3.1% (1/32)	0.0% (0/32)	44.1% (26/59)	44.1% (26/59)	10.2% (6/59)	1.7% (1/59)	0.0% (0/59)	0.4936
USR5	0.0% (0/91)	4.4% (4/91)	8.8% (8/91)	53.9% (49/91)	33.0% (30/91)	0.0% (0/32)	3.1% (1/32)	0.0% (0/32)	53.1% (17/32)	43.8% (14/32)	0.0% (0/59)	5.1% (3/59)	13.6% (8/59)	54.2% (32/59)	27.1% (16/59)	0.0994
USR6	73.6% (67/91)	25.3% (23/91)	0.0% (0/91)	1.1% (1/91)	0.0% (0/91)	81.3% (26/32)	18.8% (6/32)	0.0% (0/32)	0.0% (0/32)	0.0% (0/32)	69.5% (41/59)	28.8% (17/59)	0.0% (0/59)	1.7% (1/59)	0.0% (0/59)	0.4141
USR7	73.6% (67/91)	25.3% (23/91)	1.1% (1/91)	0.0% (0/91)	0.0% (0/91)	78.1% (25/32)	21.9% (7/32)	0.0% (0/32)	0.0% (0/32)	0.0% (0/32)	71.2% (42/59)	27.1% (16/59)	1.7% (1/59)	0.0% (0/59)	0.0% (0/59)	0.6364

The opinions of 2nd year medical students were compared with those of 4th and 6th year. *p* < 0.05 was considered statistically significant. TA, Totally agree; A, Agree; N, Neither agree nor disagree; D, Disagree; TD, Totally disagree. USR1: *I consider that participation in a research project during medical school is important*; USR2: *I consider that professors encourage us enough to participate in scientific research activities;* USR3: *The end-of-degree project (EDP) should be a research project;* USR4: *Research allows an adequate understanding of the methods applied in clinical studies;* USR5: *I consider that medical students should not participate as researchers in scientific studies;* USR6: *I consider that scientific research has a relevant role in the field of medicine;* USR7: *Patient outcomes improve with ongoing medical research.*

The vast majority of the participants think that medical students should participate in scientific studies, as they consider that scientific research plays a relevant role in the field of medicine (USR6), allowing an adequate understanding of the methods applied in the clinical studies (USR4) and improving patient outcomes (USR7).

Furthermore, 73.6% (67/91) of medical students consider participation in a research project during medical school to be important (USR1) and only 5.5% (5/91) of medical students voted the opposite. It is worth noting that these 5 students are students in 4th and 6th year of university course. In addition, 2nd year medical students had much more initiative to carry out a research project for the end-of-degree project (EDP) than 4th and 6th year medical students (*p* < 0.0001).

**Figure 2:**
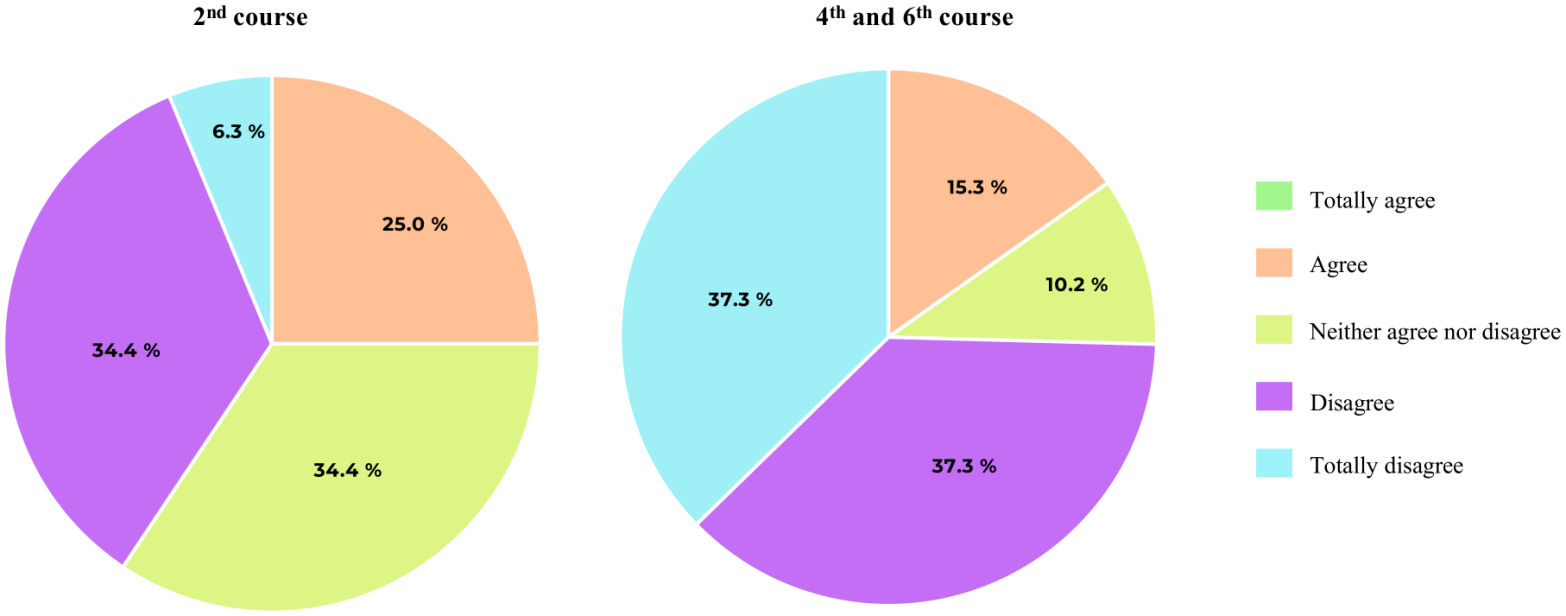
Pie chart representation of medical students’ votes on item USR2 (*I consider that professors encourage us enough to participate in scientific research activities*).

Finally, 62.6% (57/91) of the students consider that professors do not encourage them enough to participate in research activities (USR2). In this question we found significant differences between courses (Figure 2). More 4th and 6th year medical students (44/59) feel that they are not motivated enough to do research than 2nd year students (13/32) (74.6% vs. 40.7%, *p* = 0.017).

### 3.3. Scientific Research in the Professional Future

In order to know the scientific vocations regarding the professional future of UGR medical students, a section of 9 (SRPF1-9) related questions was included (Table 2). Questions about interest in participating in projects, clinical trials, doing a doctoral thesis as well as finding out about grants and funding opportunities were included.

**Table 2:** Survey section 2: Scientific Research in the Professional Future, composed of 9 items (SRPF1-9).

Item	Total					2nd Course					4th and 6th Course					*p* value
	TA	A	N	D	TD	TA	A	N	D	TD	TA	A	N	D	TD	
SRPF1	28.6% (26/91)	49.5% (45/91)	17.6% (16/91)	3.3% (3/91)	1.1% (1/91)	37.5% (12/32)	53.1% (17/32)	6.3% (2/32)	3.1% (1/32)	0.0% (0/32)	23.7% (14/59)	47.5% (28/59)	23.7% (14/59)	3.4% (2/59)	1.7% (1/59)	0.2257
SRPF2	15.4% (14/91)	51.7% (47/91)	24.2% (22/91)	6.6% (6/91)	2.2% (2/91)	25.0% (8/32)	53.1% (17/32)	15.6% (5/32)	3.1% (1/32)	3.1% (1/32)	10.2% (6/59)	50.9% (30/59)	28.8% (17/59)	8.5% (5/59)	1.7% (1/59)	0.2334
SRPF3	8.8% (8/91)	24.2% (22/91)	31.9% (29/91)	23.1% (21/91)	12.1% (11/91)	6.3% (2/32)	18.8% (6/32)	37.5% (12/32)	25.0% (8/32)	12.5% (4/32)	10.2% (6/59)	27.1% (16/59)	28.8% (17/59)	22.0% (13/59)	11.9% (7/59)	0.8194
SRPF4	23.1% (21/91)	37.4% (34/91)	28.6% (26/91)	6.6% (6/91)	4.4% (4/91)	31.3% (10/32)	46.9% (15/32)	12.5% (4/32)	3.1% (1/32)	6.3% (2/32)	18.6% (11/59)	32.2% (19/59)	37.3% (22/59)	8.5% (5/59)	3.4% (2/59)	0.0576
SRPF5	12.1% (11/91)	30.8% (28/91)	28.6% (26/91)	23.1% (21/91)	5.5% (5/91)	25.0% (8/32)	31.3% (10/32)	18.8% (6/32)	21.9% (7/32)	3.1% (1/32)	5.1% (3/59)	30.5% (18/59)	33.9% (20/59)	23.7% (14/59)	6.8% (4/59)	0.0608
SRPF6	24.2% (22/91)	59.3% (54/91)	12.1% (11/91)	4.4% (4/91)	0.0% (0/91)	31.3% (10/32)	62.5% (20/32)	6.3% (2/32)	0.0% (0/32)	0.0% (0/32)	20.3% (12/59)	57.6% (34/59)	15.3% (9/59)	6.8% (4/59)	0.0% (0/59)	0.1980
SRPF7	45.1% (41/91)	45.1% (41/91)	8.8% (8/91)	1.1% (1/91)	0.0% (0/91)	59.4% (19/32)	31.3% (10/32)	9.4% (3/32)	0.0% (0/32)	0.0% (0/32)	37.3% (22/59)	52.5% (31/59)	8.5% (5/59)	1.7% (1/59)	0.0% (0/59)	0.1796
SRPF8	22.0% (20/91)	37.7% (34/91)	26.4% (24/91)	8.8% (8/91)	5.5% (5/91)	37.5% (12/32)	37.5% (12/32)	18.8% (6/32)	0.0% (0/32)	6.3% (2/32)	13.6% (8/59)	37.3% (22/59)	30.5% (18/59)	13.6% (8/59)	5.1% (3/59)	0.0278
SRPF9	51.7% (47/91)	44.0% (40/91)	4.4% (4/91)	0.0% (0/91)	0.0% (0/91)	68.8% (22/32)	31.3% (10/32)	0.0% (0/32)	0.0% (0/32)	0.0% (0/32)	42.4% (25/59)	50.9% (30/59)	6.8% (4/59)	0.0% (0/59)	0.0% (0/59)	0.0338

The opinions of 2nd year medical students were compared with those of 4th and 6th year. *p* < 0.05 was considered statistically significant. TA, Totally agree; A, Agree; N, Neither agree nor disagree; D, Disagree; TD, Totally disagree. SRPF1: *I consider participating in research projects during my career;* SRPF2: *I am interested in understanding the basics of the scientific research process;* SRPF3: *I wish to pursue a career as an university professor in the future;* SRPF4: *I am interested in improving opportunities to be selected in a call for research related grants or fellowships;* SRPF5: *I have an interest in writing and reviewing scientific papers;* SRPF6: *I am interested in participating in clinical trials;* SRPF7: *I am interested in participating in regional. National and international congresses (both passively and actively);* SRPF8: *I am interested in doing a doctoral thesis;* SRPF9: *I believe that participation in research projects makes you grow as a medical professional.*

Most of the students showed interest in participating in research projects (SRPF1), and agreed on the importance of research in understanding clinical studies (SRPF2). The participants showed particular interest in participating in clinical trials (SRPF6) and attending congresses (SRPF7). Regarding their interest in their training as university professors, 35.2% of the students showed no interest (32/91) and 31.9% of the participants gave a neutral response (29/91) (SRPF3).

**Figure 3:**
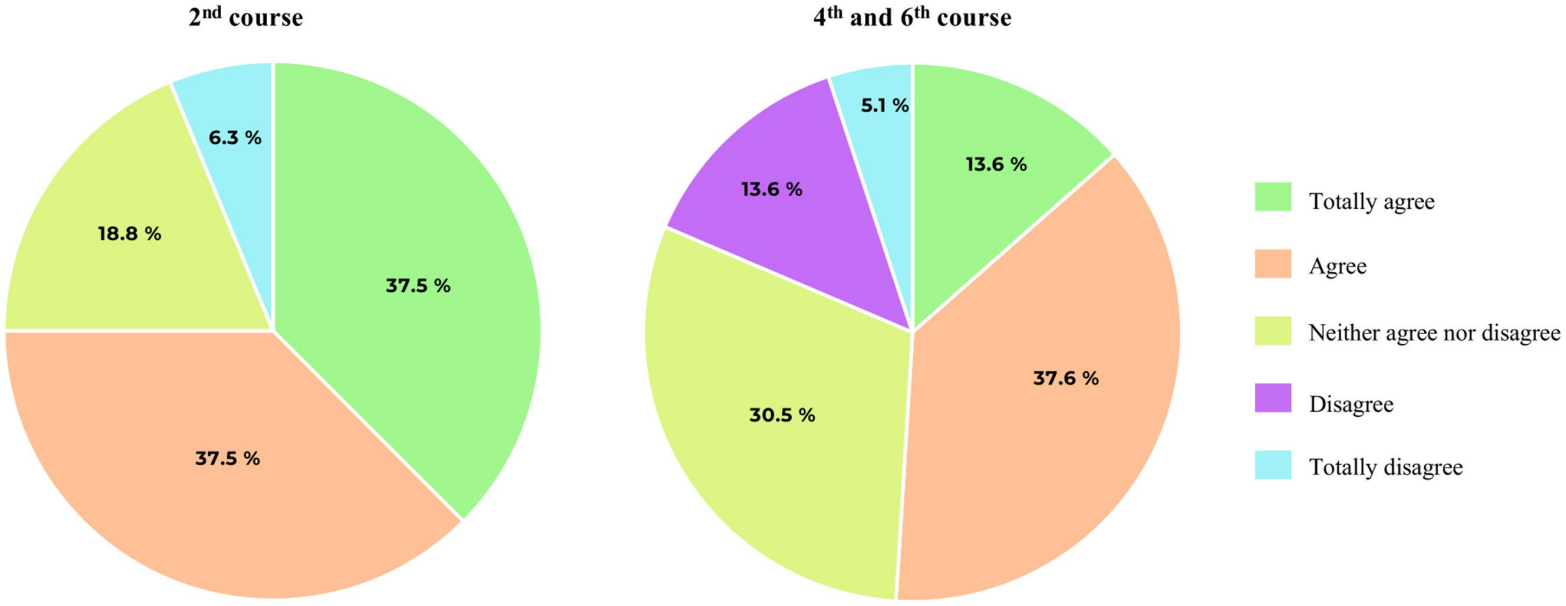
Pie chart representation of medical students’ votes on item SRPF8 (*I am interested in doing a doctoral thesis*).

The 2nd year students showed more interest in participating in calls for research grants and scholarships (78.2% vs. 50.8%) (SRPF4, *p* = 0.0576) and in writing and reviewing scientific articles (56.3% vs. 35.6%) (SRPF5, *p* = 0.0608) than the 4th and 6th year students. Moreover, 2nd year medical students have higher interest in doing a doctoral thesis (SRPF8) than 4th and 6th year students (75.4% vs. 50.9%, *p* = 0.0278) (Figure 3).

### 3.4. Participation in research activities

To identify students’ initiative concerning collaboration, communication and scientific dissemination, a section of 4 specific questions (PRA4) was included (Supplementary Table S2). To find out the level of awareness and participation of medical students in scientific dissemination activities, students were asked about two of the most popular scientific events among researchers in Europe, such as the European Science Week, held since 1993, and the European Researchers’ Night, which takes place simultaneously in almost 400 European cities.

71.5% (65/91) of medical students had not participated in faculty committees (PRA1) and 68.2% (62/91) had not participate in science promotion events such as “Science Week” (PRA3) nor 69.3% (63/91) in the “European Researchers’ Night” (PRA4).

Regarding attendance and works presentation congresses (PRA2), participation was less than 20.0%. Participation was especially low among 2nd year students (*p* = 0.287).

### 3.5. Barriers to scientific research

To determine the barriers identified by UGR medical students, a section of 7 questions (BSR1-7) was included in the survey (Supplementary Table S3).

The main barriers identified by students were the lack of research funding (BSR7) and lack of knowledge of opportunities to access research careers (BSR6) (Table 3). A significant percentage of students believe that research may decrease academic performance (BSR1), take time away from family leisure (BSR2) and delay the time to practice as a physician (BSR3). Interestingly, 2nd year medical students feel they have the right tools to engage in research, while 4th and 6th year medical students do not believe they do (56.3% vs. 28.8%) (BSR4, *p* = 0.0043).

**Table 3:** Barriers to engaging in scientific research identified by medical students.

Item	Frequency
BSR1. *Poor academic performance*	19.8% (18/91)
BSR2. *Less leisure time*	27.5% (25/91)
BSR3. *Delays the time to practice as a physician*	23.1% (21/91)
BSR4. *Lack of skills*	40.7% (37/91)
BSR5. *Increased overwhelm*	17.6% (16/91)
BSR6. *Ignorance of opportunities*	80.3% (73/91)
BSR7. *Low funding*	97.8% (89/91)

### 3.6. Expectation values and self-perceived skills

To assess the expectation values of medical students regarding scientific research and the self-perceived competences related to scientific databases, scientific publications, and tools for referencing papers, a section of 7 questions (EVSPS1-7) was included (Supplementary Table S4).

Students have a positive opinion regarding scientific papers and their relevance in their training (EVSPS1-2). In addition, 93.5% (85/91) of the students consider that research can improve organizational and/or teamwork skills (EVSPS3) and all of them agreed that research is necessary for the advancement of medicine (EVSPS4).

In relation to self-perceived skills, most medical students know how to extract the information efficiently from scientific publications (EVSPS5) and have the ability to search on a specific topic in scientific databases (EVSPS6). However, they do not know the tools to reference papers in a proper format (EVSPS7).

## 4. Discussion

This study provides a unique insight into the changing trends in the scientific vocations of medical students during their university education.

Nowadays, the interest of medical students in scientific research is controversial. Most studies claim that interest in undergraduate research activities has grown (Ferreira amorim (9)), as well as that the number of papers authored by medical students has increased in recent years (10). Furthermore, in the study of Fouad et al., students highly cited engagement to research as one of the main reasons for doing international training (11). Controversially, other studies point out that the status of undergraduate research is unsatisfactory and less static (12), that the number of physician scientists has declined over the past two decades (13) and that despite the increase in scientific publications, most of these have not been cited (10).

Our results show that medical students are aware that research plays an essential role in medical advancement. About 75.0% of the surveyed students think that it would be interesting to participate in research projects during the university degree, and about 50.0% that the EDP should be a research project. Therefore, medical students showed interest in getting involved in research during undergraduate studies, with slightly more 2nd year students being interested in this task.

Medical professors promote the inclusion of research into medical students training in order to provide them with up-to-date knowledge, foster critical and analytical skills, develop communication skills and lay the foundation for a future career in medical research. However, from the students’ point of view, they do not feel that they are sufficiently encouraged to engage in scientific research, especially students in 4th and 6th course. This fact could be related to students’ lack of awareness of opportunities to access research, the second major barrier voted by students in our survey. Perhaps there is a stronger focus on clinical theory than on research, as well as a lack of motivation for research in the classrooms. This issue can be achieved through mentoring programs, early exposure to research, integration of research into the curriculum, and promotion of appropriate incentives and recognition. In this way, more medical students could be encouraged to participate in research activities.

Regarding to the professional future, most of the students surveyed showed no interest in pursuing a doctoral thesis, especially students in their 4th and 6th year of course. These are students with superior clinical knowledge, who have taken clinical subjects, which could explain their special interest in learning and dedicating themselves to clinical practice instead of scientific research. However, medicine and research should not be seen as different paths, as these areas that should be combined and have a common goal: patients’ care improvement and advance in clinical outcomes.

The participation of medical students in scientific research activities was very scarce. Medical student participation in research is associated with an improvement in scientific productivity, more informed career choices and improved interest and attitudes towards research (14). However, around 70.0% of the medical students surveyed were not aware of and had not participated in the European Science Week (15), nor in the European Researchers’ Night (16), two of the most widespread scientific events in Europe. Encouraging medical students to get involved in scientific events can enhance their curriculum, open collaboration paths and networking, broaden their knowledge and keep them updated.

Concerning the main barriers for research, most students thought that the most important ones were the lack funding and opportunities. This is consistent with other studies that have reported limited resources and lack of funding as blockades to research involvement (2, 17, 18). Lack of awareness of opportunities was also consistent with a previous study (17, 19). In this sense, scientific committees, conferences, and science outreach events are essential sources of relevant scientific information where students could approach opportunities. In addition, strategies such as Scholarly Research Projects or Scholarly Research Concentrations could help students target their scientific interests.

Surprisingly, more 2nd year students believe they have the skills to engage in research as opposed to 4th and 6th year students, likely because during the medical degree years they have oriented their training towards clinical subjects. Previous studies also describe a decline in interest in participation in scientific research at higher levels of medical school (20). Almost ¼ of the student thought that the lack of leisure time and the delay in practicing as a physician were also barriers for research.

Students’ attitudes toward health research decreased with increasing years of medical school training. A greater number of 2nd year students consider that reading publications is important for their training and that it improves teamwork skills. In addition, 4th and 6th year students are more frustrated reading scientific articles compared to 2nd year medical students. This idea may be closely related to the model of medical studies in Europe, where students, once they have completed their basic studies, sit a competitive entrance examination and are ranked according to their marks. Only the students with the best marks will be able to access medical studies. The duration of medical careers in Europe is typically 6 years with no gap years, with the first 2–3 years focusing on basic sciences and medical theory. As they progress through their studies, students begin to participate in clinical experiences in hospitals. Probably due to this design, 4th and 6th year students feel more detached from research than students starting their medical studies.

In addition, respondents showed self-perceived skills in reading and understanding scientific articles, but did not know how to use tools to adequately reference them. Mandatory participation in research activities and scientifically oriented courses improve students’ knowledge and attitudes towards research (21) and could change students’ perceptions towards research (22). Multiple studies have shown that research methodology courses early in the curriculum can increase students’ interest in pursuing a research career (23). Thus, there is an urgent need to include mentoring programs in medical studies, expand research methodology courses, and offer students the opportunity to do research. Motivation systems, such as scholarships and international exchanges, should be offered to those conducting high-quality research to extend their opportunities in the professional future. Attention should also be given to students who are not interested in research to provide them with the basics of methodology and scientific publishing that could be useful in their clinical career.

### 4.1. Strengths and limitations of the study

The QR code survey was an efficient and scalable way to collect data from a large number of participants. The use of structured and standardized items ensures consistency of responses, facilitating the statistical analysis of the data collected. By comparing the results of students in different academic years we were able to analyze changes in attitudes, opinions and perspectives throughout their university studies. Furthermore, preserving the anonymity and confidentiality of survey participants encourages more honest and candid responses.

Regarding limitations, this is a cross-sectional, single center study that could not represent the state of the art of all the universities in Spain. Nevertheless, the Faculty of Medicine of the UGR has a very high yearly admission rate and includes students from nearly all Spanish provinces with a variety of backgrounds.

## 5. Conclusion

Medical students show a good perception about the need for research but scarce interest in participating in an investigation. Moreover, students that had taken clinical subjects (4th and 6th year students) were less attracted to investigate than those that had not taken clinical subjects (2nd year students). A lack of encouragement on research during their degree or a tendency to separate research from clinical practice could be the main reasons. Measures should be taken to improve students’ interest in research during their university studies.

## Data availability statement

The raw data supporting the conclusions of this article will be made available by the authors, without undue reservation.

## Ethics statement

This study were conducted in accordance with the local legislation and institutional requirements and was approved by the Committee on Ethics in Human Research UGR (CEIH-UGR) under the number 3138/CEIH/2023, dated 30 January 2023. The nature of the study was explained to all participants, who agreed to participate by giving their written electronic consent. The measurements were non-invasive, and the confidentiality of participants’ data was strictly preserved.

## Author contributions

RS-d: Conceptualization, Data curation, Investigation, Methodology, Visualization, Writing – original draft, Writing – review & editing. MQ-V: Conceptualization, Methodology, Writing – review & editing. AU-R: Conceptualization, Methodology, Writing – review & editing. AB-E: Project administration, Resources, Validation, Writing – review & editing. TM-V: Conceptualization, Data curation, Formal analysis, Software, Supervision, Visualization, Writing – review & editing. SA-S: Conceptualization, Funding acquisition, Project administration, Resources, Supervision, Validation, Visualization, Writing – review & editing.
