# Supplementary material for: Medical students’ interest in research: changing trends during university training
Source: Front Med (Lausanne). 2023 Oct 19;10:1257574. doi: 10.3389/fmed.2023.1257574 (PMC10620684; doi:10.3389/fmed.2023.1257574)
Supplement: Supplementary file 1 [file Data_Sheet_1.docx]

Supplementary Material

## Supplementary Table 1

| *Section* | Item | Question | TA | A | N | D | TD |
| --- | --- | --- | --- | --- | --- | --- | --- |
| *Undergraduate Scientific Research* | USR1 | I consider that participation in a research project during medical school is important. |  |  |  |  |  |
|  | USR2 | I consider that professors encourage us enough to participate in scientific research activities. |  |  |  |  |  |
|  | USR3 | The end-of-degree project (EDP) should be a research project. |  |  |  |  |  |
|  | USR4 | Research allows an adequate understanding of the methods applied in clinical studies. |  |  |  |  |  |
|  | USR5 | I consider that medical students should not participate as researchers in scientific studies. |  |  |  |  |  |
|  | USR6 | I consider that scientific research has a relevant role in the field of medicine. |  |  |  |  |  |
|  | USR7 | Patient outcomes improve with ongoing medical research. |  |  |  |  |  |
| *Scientific Research in the Professional Future* | SRPF1 | I consider participating in research projects during my career. |  |  |  |  |  |
|  | SRPF2 | I am interested in understanding the basics of the scientific research process. |  |  |  |  |  |
|  | SRPF3 | I wish to pursue a career as an university professor in the future. |  |  |  |  |  |
|  | SRPF4 | I am interested in improving opportunities to be selected in a call for research related grants or fellowships. |  |  |  |  |  |
|  | SRPF5 | I have an interest in writing and reviewing scientific papers. |  |  |  |  |  |
|  | SRPF6 | I am interested in participating in clinical trials. |  |  |  |  |  |
|  | SRPF7 | I am interested in participating in regional. national and international congresses (both passively and actively). |  |  |  |  |  |
|  | SRPF8 | I am interested in doing a doctoral thesis. |  |  |  |  |  |
|  | SRPF9 | I believe that participation in research projects makes you grow as a medical professional. |  |  |  |  |  |
| *Participation in Research Activities* | PRA1 | I have participated in medical school committees. |  |  |  |  |  |
|  | PRA2 | I have presented works at conferences and seminars (talks and posters) during my career. |  |  |  |  |  |
|  | PRA3 | I am aware of the ''Science Week'' event and have attended or participated. |  |  |  |  |  |
|  | PRA4 | I am familiar with the event ''European Researchers' Night'' and have attended or participated. |  |  |  |  |  |
| *Barriers to Scientific Research* | BSR1 | I consider that investing time in research during my career is detrimental to my academic performance by taking away from study time. |  |  |  |  |  |
|  | BSR2 | I consider that research reduces the time available for family leisure, and therefore, it does not interest me. |  |  |  |  |  |
|  | BSR3 | I consider that research increases training time, and therefore, delays the time to practice as a physician. |  |  |  |  |  |
|  | BSR4 | I consider that I do not have the appropriate skills to engage in research. |  |  |  |  |  |
|  | BSR5 | I consider that introducing research during the medical degree is unnecessary because it increases the pressure on students. |  |  |  |  |  |
|  | BSR6 | I consider that medical students are not aware of the opportunities to access research. |  |  |  |  |  |
|  | BSR7 | I consider that in Spain the investment in research is not sufficient. |  |  |  |  |  |
| *Expectation Values and*  *Self-Perceived Skills* | EVSPS1 | I find reading scientific papers frustrating. |  |  |  |  |  |
|  | EVSPS2 | I consider that reading scientific papers is important for my training. |  |  |  |  |  |
|  | EVSPS3 | I consider that working in research improves organizational and/or teamwork skills. |  |  |  |  |  |
|  | EVSPS4 | I consider that research is necessary for the advancement of medicine. |  |  |  |  |  |
|  | EVSPS5 | I consider that I am able to extract information efficiently from scientific papers. |  |  |  |  |  |
|  | EVSPS6 | I consider that I know the tools to search for scientific papers on a specific topic. |  |  |  |  |  |
|  | EVSPS7 | I consider that I know how to reference scientific papers correctly (APA or Vancouver format). |  |  |  |  |  |

**Supplementary Table 1.** English version of the survey distributed to UGR medical students. The survey is divided into 5 main sections with their corresponding items: 1) Undergraduate Scientific Research (USR), 2) Scientific Research in the Professional Future (SRPF), 3) Participation in Research Activities (PRA), 4) Barriers to Scientific Research (BSR) and 5) Expectation Values and Self-Perceived Skills (EVSPS). TA: Totally agree; A: Agree; N: Neither agree nor disagree; D: Disagree; TD: Totally disagree.

## Supplementary Table 2

| **Item** | **Total** | | | | | **2^nd^ course** | | | | | **4^th^ and 6^th^ course** | | | | | **p value** |
| --- | --- | --- | --- | --- | --- | --- | --- | --- | --- | --- | --- | --- | --- | --- | --- | --- |
|  | **TA** | **A** | **N** | **D** | **TD** | **TA** | **A** | **N** | **D** | **TD** | **TA** | **A** | **N** | **D** | **TD** |  |
| PRA1 | 4.4% (4/91) | 11.0% (10/91) | 13.2% (12/91) | 35.2% (32/91) | 36.3% (33/91) | 6.3% (2/32) | 15.6% (5/32) | 9.4% (3/32) | 25.0% (8/32) | 43.8% (14/32) | 3.4% (2/59) | 8.5% (5/59) | 15.3% (9/59) | 40.7% (24/59) | 32.2% (19/59) | 0.3916 |
| PRA2 | 2.2% (2/91) | 15.4% (14/91) | 5.5% (5/91) | 33.0% (30/91) | 44.0% (40/91) | 0.0% (0/32) | 6.3% (2/32) | 9.4% (3/32) | 21.9% (7/32) | 62.5% (20/32) | 3.4% (2/59) | 20.3% (12/59) | 3.4% (2/59) | 39.0% (23/59) | 33.9% (20/59) | 0.0287 |
| PRA3 | 6.6% (6/91) | 20.9% (19/91) | 4.4% (4/91) | 35.2% (32/91) | 33.0% (30/91) | 3.1% (1/32) | 25.0% (8/32) | 3.1% (1/32) | 37.5% (12/32) | 31.3% (10/32) | 8.5% (5/59) | 18.6% (11/59) | 5.1% (3/59) | 33.9% (20/59) | 33.9% (20/59) | 0.8081 |
| PRA4 | 7.7% (7/91) | 15.4% (14/91) | 7.7% (7/91) | 31.9% (29/91) | 37.4% (34/91) | 3.1% (1/32) | 15.6% (5/32) | 12.5% (4/32) | 31.3% (10/32) | 37.5% (12/32) | 10.2% (6/59) | 15.3% (9/59) | 5.1% (3/59) | 32.2% (19/59) | 37.3% (22/59) | 0.5867 |

**Supplementary Table 2.** Survey Section 3: Participation in Research Activities, composed of 4 items (PRA1-4). The opinions of 2^nd^ year medical students were compared with those of 4^th^ and 6^th^ year. p < 0.05 was considered statistically significant. TA: Totally agree; A: Agree; N: Neither agree nor disagree; D: Disagree; TD: Totally disagree. PRA1: *I have participated in medical school committees;* PRA2: *I have presented works at conferences and seminars (talks and posters) during my career;* PRA3: *I am aware of the ''Science Week'' event and have attended or participated;* PRA4: *I am familiar with the event ''European Researchers' Night'' and have attended or participated*.

## Supplementary Table 3

| **Item** | **Total** | | | | | **2^nd^ course** | | | | | **4^th^ and 6^th^ course** | | | | | **p value** |
| --- | --- | --- | --- | --- | --- | --- | --- | --- | --- | --- | --- | --- | --- | --- | --- | --- |
|  | **TA** | **A** | **N** | **D** | **TD** | **TA** | **A** | **N** | **D** | **TD** | **TA** | **A** | **N** | **D** | **TD** |  |
| BSR1 | 5.5% (5/91) | 14.3% (13/91) | 18.7% (17/91) | 33.0% (30/91) | 28.6% (26/91) | 3.1% (1/32) | 6.3% (2/32) | 15.6% (5/32) | 37.5% (12/32) | 37.5% (12/32) | 6.8% (4/59) | 18.6% (11/59) | 20.3% (12/59) | 30.5% (18/59) | 23.7% (14/59) | 0.3232 |
| BSR2 | 8.8% (8/91) | 18.7% (17/91) | 16.5% (15/91) | 39.6% (36/91) | 16.5% (15/91) | 6.3% (2/32) | 15.6% (5/32) | 12.5% (4/32) | 34.4% (11/32) | 31.3% (10/32) | 10.2% (6/59) | 20.3% (12/59) | 18.6% (11/59) | 42.4% (25/59) | 8.5% (5/59) | 0.0935 |
| BSR3 | 2.2% (2/91) | 20.9% (19/91) | 20.9% (19/91) | 44.0% (40/91) | 12.1% (11/91) | 3.1% (1/32) | 18.8% (6/32) | 15.6% (5/32) | 40.6% (13/32) | 21.9% (7/32) | 1.7% (1/59) | 22.0% (13/59) | 23.7% (14/59) | 45.8% (27/59) | 6.8% (4/59) | 0.2885 |
| BSR4 | 12.1% (11/91) | 28.6% (26/91) | 20.9% (19/91) | 27.5% (25/91) | 11.0% (10/91) | 12.5% (4/32) | 6.3% (2/32) | 25.0% (8/32) | 34.4% (11/32) | 21.9% (7/32) | 11.9% (7/59) | 40.7% (24/59) | 18.6% (11/59) | 23.7% (14/59) | 5.1% (3/59) | 0.0043 |
| BSR5 | 5.5% (5/91) | 12.1% (11/91) | 14.3% (13/91) | 35.2% (32/91) | 33.0% (30/91) | 3.1% (1/32) | 9.4% (3/32) | 6.3% (2/32) | 43.8% (14/32) | 37.5% (12/32) | 6.8% (4/59) | 13.6% (8/59) | 18.6% (11/59) | 30.5% (18/59) | 30.5% (18/59) | 0.3573 |
| BSR6 | 28.6% (26/91) | 51.7% (47/91) | 14.3% (13/91) | 4.4% (4/91) | 1.1% (1/91) | 21.9% (7/32) | 50.0% (16/32) | 21.9% (7/32) | 3.1% (1/32) | 3.1% (1/32) | 32.2% (19/59) | 52.5% (31/59) | 10.2% (6/59) | 5.1% (3/59) | 0.0% (0/59) | 0.3068 |
| BSR7 | 68.1% (62/91) | 29.7% (27/91) | 2.2% (2/91) | 0.0% (0/91) | 0.0% (0/91) | 81.3% (26/32) | 15.6% (5/32) | 3.1% (1/32) | 0.0% (0/32) | 0.0% (0/32) | 61.0% (36/59) | 37.3% (22/59) | 1.7% (1/59) | 0.0% (0/59) | 0.0% (0/59) | 0.0944 |

**Supplementary Table 3.** Survey Section 4: Barriers to Scientific Research, composed of 7 items (BSR1-7). The opinions of 2^nd^ year medical students were compared with those of 4^th^ and 6^th^ year. p < 0.05 was considered statistically significant. TA: Totally agree; A: Agree; N: Neither agree nor disagree; D: Disagree; TD: Totally disagree. BSR1: *I consider that investing time in research during my career is detrimental to my academic performance by taking away from study time;* BSR2: *I consider that research reduces the time available for family leisure, and therefore, it does not interest me;* BSR3: *I consider that research increases training time, and therefore, delays the time to practice as a physician;* BSR4: *I consider that I do not have the appropriate skills to engage in research;* BSR5: *I consider that introducing research during the medical degree is unnecessary because it increases the pressure on students;* BSR6: *I consider that medical students are not aware of the opportunities to access research;* BSR7: *I consider that in Spain the investment in research is not sufficient.*

## Supplementary Table 4

| **Item** | **Total** | | | | | **2^nd^ course** | | | | | **4^th^ and 6^th^ course** | | | | | **p value** |
| --- | --- | --- | --- | --- | --- | --- | --- | --- | --- | --- | --- | --- | --- | --- | --- | --- |
|  | **TA** | **A** | **N** | **D** | **TD** | **TA** | **A** | **N** | **D** | **TD** | **TA** | **A** | **N** | **D** | **TD** |  |
| EVSPS1 | 8.8% (8/91) | 23.1% (21/91) | 27.5% (25/91) | 35.2% (32/91) | 5.5% (5/91) | 9.4% (3/32) | 18.8% (6/32) | 21.9% (7/32) | 40.6% (13/32) | 9.4% (3/32) | 8.5% (5/59) | 25.4% (15/59) | 30.5% (18/59) | 32.2% (19/59) | 3.4% (2/59) | 0.5999 |
| EVSPS2 | 36.3% (33/91) | 48.4% (44/91) | 12.1% (11/91) | 2.2% (2/91) | 1.1% (1/91) | 43.8% (14/32) | 46.9% (15/32) | 6.3% (2/32) | 3.1% (1/32) | 0.0% (0/32) | 32.2% (19/59) | 49.2% (29/59) | 15.3% (9/59) | 1.7% (1/59) | 1.7% (1/59) | 0.5727 |
| EVSPS3 | 51.7% (47/91) | 41.8% (38/91) | 5.5% (5/91) | 1.1% (1/91) | 0.0% (0/91) | 59.4% (12/32) | 37.5% (12/32) | 3.1% (1/32) | 0.0% (0/32) | 0.0% (0/32) | 47.5% (28/59) | 44.1% (26/59) | 6.8% (4/59) | 1.7% (1/59) | 0.0% (0/59) | 0.6081 |
| EVSPS4 | 90.1% (82/91) | 9.9% (9/91) | 0.0% (0/91) | 0.0% (0/91) | 0.0% (0/91) | 93.8% (30/32) | 6.3% (2/32) | 0.0% (0/32) | 0.0% (0/32) | 0.0% (0/32) | 88.1% (52/59) | 11.9% (7/59) | 0.0% (0/59) | 0.0% (0/59) | 0.0% (0/59) | 0.3916 |
| EVSPS5 | 7.7% (7/91) | 49.5% (45/91) | 30.8% (28/91) | 11.0% (10/91) | 1.1% (1/91) | 12.5% (4/32) | 43.8% (14/32) | 37.5% (12/32) | 6.3% (2/32) | 0.0% (0/32) | 5.1% (3/59) | 52.5% (31/59) | 27.1% (16/59) | 13.6% (8/59) | 1.7% (1/59) | 0.3946 |
| EVSPS6 | 8.8% (8/91) | 44.0% (40/91) | 23.1% (21/91) | 22.0% (20/91) | 2.2% (2/91) | 9.4% (3/32) | 34.4% (11/32) | 28.1% (9/32) | 25.0% (8/32) | 3.1% (1/32) | 8.5% (5/59) | 49.2% (29/59) | 20.3% (12/59) | 20.3% (12/59) | 8.5% (5/59) | 0.7370 |
| EVSPS7 | 8.8% (8/91) | 28.6% (26/91) | 19.8% (18/91) | 24.2% (22/91) | 18.7% (17/91) | 6.3% (2/32) | 15.6% (5/32) | 12.5% (4/32) | 37.5% (12/32) | 28.1% (9/32) | 10.2% (6/59) | 35.6% (21/59) | 23.7% (14/59) | 17.0% (10/59) | 13.6% (8/59) | 0.0320 |

**Supplementary Table 4.** Survey Section 5: Expectation Values and Self-Perceived Skills, composed of 7 items (EVSPS1-7). The opinions of 2^nd^ year medical students were compared with those of 4^th^ and 6^th^ year. p < 0.05 was considered statistically significant. TA: Totally agree; A: Agree; N: Neither agree nor disagree; D: Disagree; TD: Totally disagree. EVSPS1: *I find reading scientific papers frustrating;* EVSPS2: *I consider that reading scientific papers is important for my training;* EVSPS3: *I consider that working in research improves organizational and/or teamwork skills;* EVSPS4: *I consider that research is necessary for the advancement of medicine;* EVSPS5: *I consider that I am able to extract information efficiently from scientific papers;* EVSPS6: *I consider that I know the tools to search for scientific papers on a specific topic;* EVSPS7: *I consider that I know how to reference scientific papers correctly (APA or Vancouver format).*
